# The Efficacy of Shen Shuaining Capsule on Chronic Kidney Disease: A Systematic Review and Meta-Analysis

**DOI:** 10.1155/2016/7515413

**Published:** 2016-02-14

**Authors:** Shanshan Wang, Jinfeng Zhang, Ming Guo, Xiaobo Lian, Miaomiao Sun, Lizhong Guo

**Affiliations:** ^1^First Clinical Medical College, Nanjing University of Chinese Medicine, Nanjing 210029, China; ^2^Chinese Medicine Department, Zhongda Hospital Southeast University, Nanjing 210000, China; ^3^Famous Doctor's Studio of Zhongying Zhou, Nanjing University of Chinese Medicine, Nanjing 210029, China

## Abstract

*Objective*. To evaluate the efficacy of Shen shuaining capsule on treating chronic kidney disease (CKD).* Methods*. All randomized controlled trials (RCTs) of Shen shuaining capsule in treating CKD were collected from CBM, CNKI, VIP, Wanfang, EMBASE, MEDLINE, PubMed, and Cochrane library clinical controlled trials database. Two reviewers independently performed analysis of the included trials according to the inclusion and exclusion criteria. The risk of bias tool was from the Cochrane Handbook version 5.1.0. The Review Manager 5.2 software was employed for data analysis. Funnel plot and Egger's test were applied to evaluate publication bias.* Results*. 20 studies including 1606 participants met the inclusion criteria, most of which were of low quality. Meta-analysis indicated that Shen shuaining capsule was effective for CKD in terms of SCR, BUN, Hb, and response rate and with less adverse effects, of which SCR and BUN decreased significantly (MD = −84.72, 95% CI: −107.36, −62.07, *P* < 0.00001) (MD = −4.30, 95% CI: −5.71, −2.89, *P* < 0.00001); Hb and response rate increased significantly (MD = 9.94, 95% CI: 9.24, 10.64, *P* < 0.00001) (OR = 4.25, 95% CI (3.32, 5.42), *P* < 0.00001).* Conclusion*. Shen shuaining capsule significantly reduced SCR and BUN, increased HB, and improved total efficiency of the symptoms and signs in patients with CKD. Subgroup analysis found that Shen shuaining capsule group was better than control group. Due to low quality of the methodology of included studies, further high-quality researches were needed to study its efficacy and safety.

## 1. Introduction

Chronic kidney disease (CKD) is a slow progressive renal damage and finally may lead to uremia and loss of renal function and causes a series of clinical symptoms and metabolic disorders such as chemical and endocrine disorders. Kidney failure is the end stage of acute and chronic kidney diseases and may require treatment by dialysis or transplantation. In China, the number of chronic dialysis patients has been increasing over the last decade [1]. Acute and chronic kidney diseases are important risk factors not only for end stage renal disease (ESRD) but also for cardiovascular disease, and they are associated with a considerable increase in morbidity and mortality [2–4]. The prevalence of CKD which increased gradually accounted for about 10.8% in China and 10-11% in both Europe and the United States [5, 6]. Though substantial progress has been made in our understanding of renal pathophysiology, there has been little progress in the way of new therapies since that time. Thus, additional intervention, including Chinese herbal medicine, may be effective for the treatment of CKD.

Traditional Chinese medicine (TCM) has been used as the main therapeutic means of diseases in China for thousands of years. Through the synergistic effect of multicomponents, multipathways, and multitargets, it showed significant advantages over a single drug treatment, especially for the treatment of chronic kidney diseases [7, 8]. Compared to Western medicine, it has been observed to have lower side effects [9–11]. Shen shuaining capsule is composed of* Rheum officinale *(*Dahuang*),* Radix pseudostellariae *(*Tai zishen*),* Coptis chinensis *(*Huanglian*),* Carthamus tinctorius (Honghua), the rhizome of Salvia miltiorrhiza *(*Danshen*), and* Bidentate achyranthes *(*Niuxi*) and it has effects of tonifying spleen Qi and kidney Qi, promoting blood circulation and removing blood stasis in TCM [12]. Chinese rhubarb,* Rheum palmatum* and* Rheum officinale*, has been used for thousands of years to treat various diseases in TCM. Chinese rhubarb and its constituents have been shown to have antifibrotic and anti-inflammatory effects* in vivo* and* in vitro* at least in part because of inhibition of TGF-*β* [13–16]. In addition, in rodent models, rhubarb supplementation has decreased proteinuria and slowed progression of kidney disease [13, 14]. These effects appear to be synergistic with concurrent renin-angiotensin aldosterone system blockade [13, 15]. Tanshinone IIA is the most abundant diterpene quinone isolated from* the rhizome of Salvia miltiorrhiza *(*Danshen*), which has been used in treating CKD for more than 2000 years in China. Tanshinone IIA has been shown to attenuate oxidative stress injury and decrease endoplasmic reticulum stress-mediated apoptosis in rat kidneys during hypothermic preservation [17]. It has been reported that Tanshinone IIA induces vasodilation and reduces blood pressure via endothelial nitric oxide synthase stimulation in renovascular hypertension model hamsters and that Tanshinone IIA has renoprotective effects on the progression of diabetic nephropathy [18, 19]. Tanshinone IIA attenuates the structural manifestations of renal disease progression and ameliorates the effects of renal dysfunction in CKD rats [20]. Evidence in the literature shows that Shen shuaining can increase the blood flow of kidney, promote the excretion of toxins, inhibit renal compensatory hypertrophy and regulate metabolic state, protect and improve the function of the remnant kidney, and delay the process [21].

At present, Shen shuaining capsule has been widely chosen for the treatment of CKD in China. However, the evidence for the effects of Shen shuaining capsule has not been systematically assessed. The aim of this study is to examine the effectiveness of Shen shuaining capsule in the treatment of CKD.

## 2. Materials and Methods

### 2.1. Data Sources and Search Strategies

Databases were searched by electronic and manual methods, including PubMed, EMBASE, the Cochrane Central Register of Controlled Trials, Web of Science, Chinese Biomedical Literature Database, China National Knowledge Infrastructure Database, Chinese Evidence-Based Medicine Database, and Wanfang Database and handsearching of reference which do not include these electronic databases noted above.

The following terms were retrieved in databases as keywords or free-text terms: “Shen shuaining,” “Shenshuaining,” “Shen shuai ning,” “Shen Shuai Ning,” “chronic renal failure,” “chronic kidney failure,” “chronic kidney disease,” and “chronic renal dysfunction.” For Chinese databases, we used free text terms as “Shan shuaining” (in Chinese), and “Man Xing Shen Shuai,” “Man Xing Shen Shuai Jie,” “Man Xing Shen Gong Neng Shuai Jie,” “Man Xing Shen Gong Neng Bu Quan,” and “Man Xing Shen Zang Bing” (in Chinese). A filter for clinical trials was applied. We also attempted to identify additional studies by searching the references list of included trials. No restrictions were placed on the publication language. Two reviewers (Shanshan Wang and Jinfeng Zhang) independently identified studies.

### 2.2. Criteria for Considering Studies

All randomized control trials (RCTs) published before May 2015 were included regardless of language. All trials reported baseline comparability. Inclusion criteria were as follows: (1) chronic renal failure (CKD) was diagnosed according to the diagnostic criteria of CKD formulated by the thematic symposium on the classification, treatment, and diagnostic criteria of primary glomerular disease [42], CKD staging criteria were proposed by K/DOQI [43], and (2) the experimental design was RCT. The literature exclusion criteria were as follows: (1) no clear diagnostic criteria for the research subjects; (2) randomization not mentioned or the experimental design not being RCT; (3) a lack of a clear course of treatment; (4) a lack of standard indicators to evaluate efficacy; (5) clinical trials of different TCMs; (6) translated from foreign literature or duplicate publications; (7) animal studies; (8) the gap of the sample size in both experimental and control groups being no more than 50%.

### 2.3. Data Extraction and Quality Assessment

The detailed method followed the reported one [44]. Two reviewers (Shanshan Wang and Jinfeng Zhang) independently extracted data. The extracted data included authors, title of study, year of publication, study size, age and sex of the participants, details of methodological information, treatment process, details of the interventions, and outcomes. Any disagreements were resolved by consensus or by a third reviewer (Lizhong Guo)

Methodological quality of RCTs was assessed independently by two review authors (Shanshan Wang and Jinfeng Zhang) with the criteria in the Cochrane Handbook for Systematic Reviews of Interventions 5.1.0 [45]. Sequence generation, allocation concealment, blinding (or masking), incomplete data assessment, selective outcome reporting, and other sources of bias were assessed with three potential responses: yes, no, and unclear. Disagreements between review authors were resolved by discussion or with the third author (Lizhong Guo).

### 2.4. Intervention Measures

Regarding the intervention measures, generally, in addition to the routine conventional treatment, ① Shen shuaining capsule treatment and placebo or no-treatment were compared; ② Shen shuaining capsule treatment and a kind of Western medicine treatment were compared (note: routine conventional treatment included the following.) (1) Protein intake (50% was high-quality protein) was 0.6 g/(kg·d) with sufficient calorie supply (30–35 kcal/kg). (2) Diuretic was used and also (3) treatment of water and electrolyte disorder,acid-base imbalance, and anemia. (4) Antihypertensive agents except ACEI or ARB were given to the patients with BP > 140/90 mmHg. (5) Patients received antihyperlipidemic agents including fenofibrate and/or atorvastatin for hyperlipoidemia. (6) Antibiotics were used in patients with infection. The capsule of Shen shuaining was prepared by Yunnan Lixiang Pharmaceutical Limited (Yunnan, China) according to the quality standard of China State Food and Drug Administration. The capsules were administrated orally. The dosage was (1.4 g–2.1 g) po tid and the treatment course ranged from 3 to 48 weeks three times a day orally.

### 2.5. Outcome Criteria

Criteria of therapeutic effects included function [blood urea nitrogen (BUN), serum creatinine (SCR)], endogenous creatinine clearance rate (CCR), hemoglobin (HB), total response rate, and adverse effects.

### 2.6. Division of Subgroups

The cases in the literature were divided into three groups, according to intervention course: (1) less than or equal to 4 weeks; (2) over 4 weeks, less than or equal to 8 weeks; and (3) over 8 weeks.

### 2.7. Statistical Methods

We conducted meta-analysis using RevMan 5.2. (Cochrane Collaboration, Oxford, UK) [46]. Estimated effect of data was calculated by standardized mean difference (SMD) or weight mean difference (WMD). Chi-square test was used for heterogeneity. We tested heterogeneity using the *I* ^2^ statistic with significance set at 50%, and the Chi^2^ statistic with significance set at *P* < 0.10. If significant heterogeneity was identified, the random-effects model was used. Trials showing clinical heterogeneity were combined according to the random effect model and the remaining studies used the fixed effect model. A statistical test of funnel plot asymmetry, which may indicate the presence of publication bias, was performed if possible [47].

## 3. Results

### 3.1. Description of Studies

**Figure 1:**
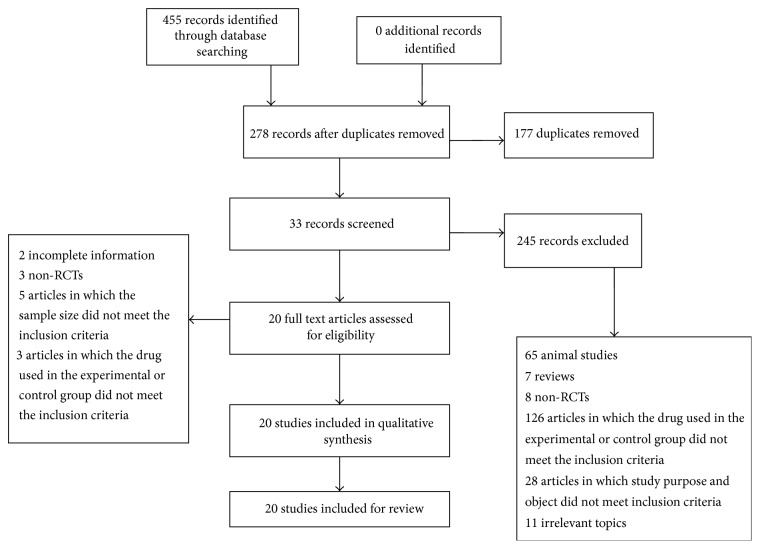
Flow diagram of the articles selection for this study.

A total of 455 articles on Shen shuaining capsule were retrieved via an online search. According to the inclusion criteria and exclusion criteria, 177 articles were ruled out due to duplication; the remaining 278 articles were carefully read to further exclude 8 non-RCTs, 65 animal studies, 7 reviews, 126 articles in which the drug used in the experimental or control group did not meet the inclusion criteria, and 28 articles in which study purpose and object did not meet inclusion criteria, 11 irrelevant topics. Finally, 20 articles were included in this review (Table 1 and Figure 1).

### 3.2. Characteristics of Included Studies

Characteristics of trials included in this review were summarized in Table 1. The included 20 trials [22–41] were conducted in China and published in Chinese, with a total of 1606 research subjects (832 of treatment group, 774 of control group). 17 trials [22, 24–33, 35, 36, 38–41] reported the number of male and female patients (838 male and 596 female), CKD patients in stages 3-4 were included in 6 studies [29–31, 33, 37, 41], and patients in stages 3–5, 2-3, and 3 were described, respectively, in 3 studies [22, 23, 34], 1 study [35], and 2 studies [38, 39].

### 3.3. Intervention Measures

All studies adopted Shen shuaining capsule plus routine conventional treatment. The course of treatment ranged from 3 weeks to 48 weeks, most at 6 weeks and 8 weeks, of which ten studies [24, 25, 28, 30, 31, 35, 37–40] compared Shen shuaining capsule plus routine conventional treatment with routine conventional treatment; nine trials [22, 23, 26, 27, 29, 33, 34, 36, 41] compared Shen shuaining capsule plus routine conventional treatment with Coated Aldehyde Oxystarch capsule plus routine conventional treatment; one trial [32] compared Shen shuaining capsule plus routine conventional treatment with Medicinal Charcoal Capsule plus routine conventional treatment.

### 3.4. Outcome Measures

Twelve studies [23, 24, 27–31, 34, 35, 37, 38, 41] adopted measurement data (SCR, BUN, CCR, and Hb) to evaluate effects, of which eight studies [24, 27–31, 34, 35] also adopted count data (total response rate). Count data (total response rate) was adopted only in the remaining eight trials [22, 25, 26, 32, 33, 36, 39, 40].

### 3.5. Assessment of Risk of Bias in Included Studies

The quality of reporting in the reviewed studies was generally poor, providing insufficient information to reach conclusions whether or not the random sequence generation, allocation concealment, and blinding were adequate. Inadequate reporting raises the possibility of bias and carries a risk for the validity of this review. Of those, only one trial [38] used random number table methods, one study [28] selected stratified randomization method, the remaining 18 studies did not mention random method (only “random” words); details on how allocation being concealed were unclear in 20 studies; none of the 20 studies described blinding of participants, personnel, and outcome assessor; no study reported the number and reasons of dropouts, loss to follow-up. We could not assess whether selective reporting or other important risks of bias existed due to insufficient information in all included studies.

### 3.6. Data Analysis

#### 3.6.1. SCR

**Figure 2:**
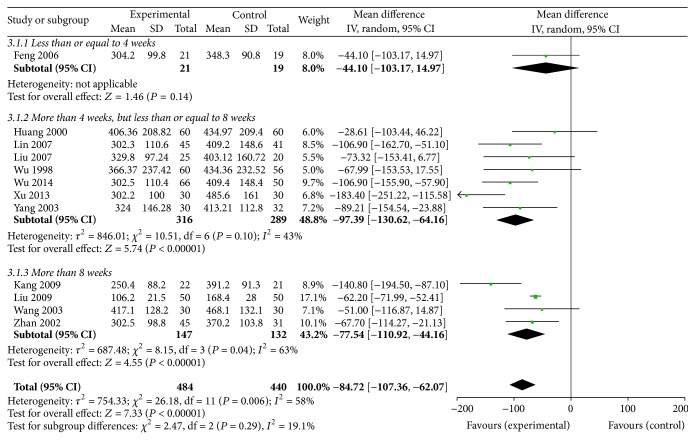
Effect of Shen shuaining capsule on serum creatinine in chronic renal failure.

12 RCTs [23, 24, 27–31, 34, 35, 37, 38, 41] evaluated the SCR of the experimental group as compared with the control group. There were 484 patients in the experimental group and 440 in the control group. The trials showed clinical heterogeneity and a random effects model was used. Significant decreases on SCR levels were observed within Shen shuaining capsule at 4–8 weeks [MD = −97.39, 95% CI (−130.62, −64.16), *P* < 0.00001], 8 weeks [MD = −77.54, 95% CI (−110.92, −44.16), *P* < 0.00001] and overall effect [MD = −84.72, 95% CI (−107.36, −62.07), *P* < 0.00001]; however, there were no significant improvements at 4 weeks [MD = −44.10, 95% CI (−103.17, 14.97), *P* = 0.14] Figure 2.

#### 3.6.2. BUN

**Figure 3:**
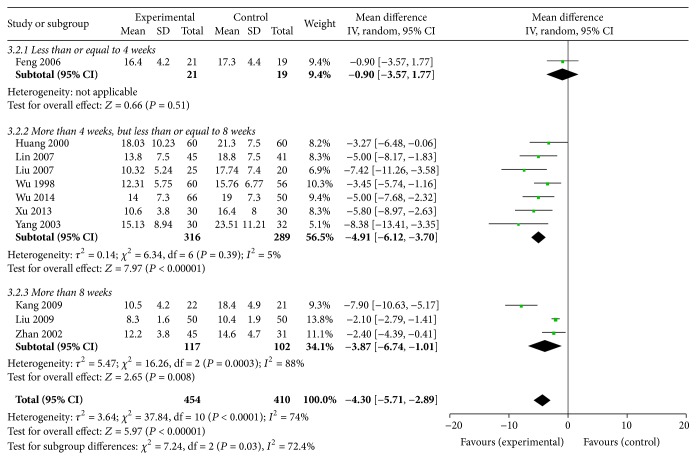
Effect of Shen shuaining capsule on blood urea nitrogen in chronic renal failure.

A total of 11 RCTs [23, 24, 27–31, 34, 35, 38, 41] (454 patients in treatment group and 410 in the control group) were conducted to analyze serum urea nitrogen. Because of significant heterogeneity, a random effects model was used. The results show that BUN levels decreased significantly in favor of Shen shuaining capsule from onset time to 4–8 weeks (MD = −4.91, 95% CI: −6.12, −3.7, *P* < 0.00001), 8 weeks (MD = −3.87, 95% CI: −6.74, −1.01, *P* = 0.008), and overall effect (MD = −4.30, 95% CI: −5.71, −2.89, *P* < 0.00001). However, there were no significant improvements at 4 weeks (MD = −0.90, 95% CI: −3.57, 1.77, *P* = 0.51), Figure 3.

#### 3.6.3. Hb

**Figure 4:**
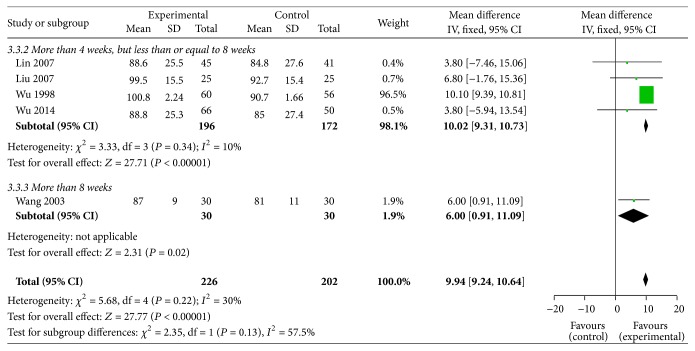
Effect of Shen shuaining capsule on hemoglobin in chronic renal failure.

5 RCTs [24, 28, 29, 37, 41] (226 patients in the treatment group and 202 in the control group) analyzed the HB. We chose the fixed effect model. The results show that the Shen shuaining group had significantly improved HB compared with the control group at 4–8 weeks (MD = 10.02, 95% CI: 9.31, 10.73, *P* < 0.00001) and 8 weeks [MD = 6.00, 95% CI (0.91, 11.09), *P* = 0.02 < 0.05] and overall effect (MD = 9.94, 95% CI: 9.24, 10.64, *P* < 0.00001), Figure 4.

#### 3.6.4. CCR

**Figure 5:**
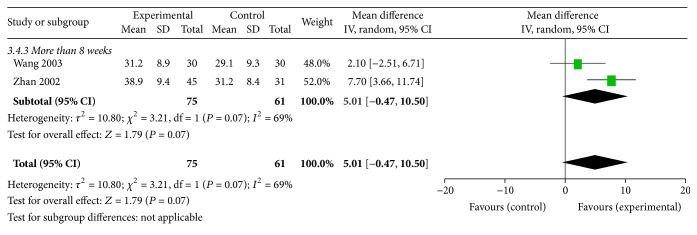
Effect of Shen shuaining capsule on endogenous creatinine clearance rate in chronic renal failure.

Two trials [34, 37] evaluated the efficacy of Shen shuaining capsule on CCR in the treatment group (75 patients) compared with the control group (61 patients). A Random effect model was used.  Figure 5 shows the forest plots for the outcome measures. There was no significant difference between the two groups (MD = 5.01, 95% CI: −0.47, 10.50, *P* = 0.07 > 0.05), Figure 5.

#### 3.6.5. Response Rate

**Figure 6:**
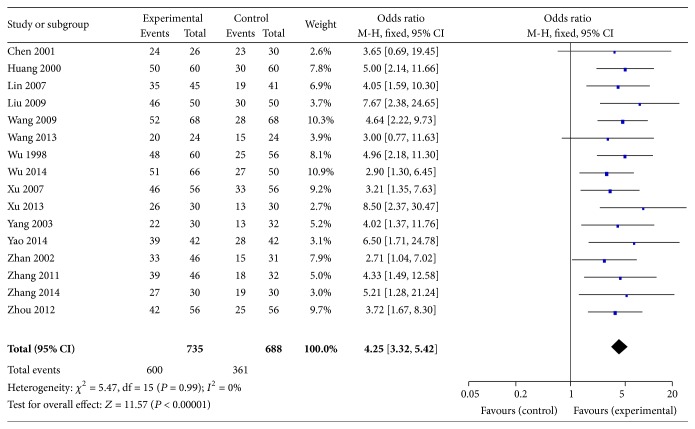
Effect of Shen shuaining capsule on total response rate in chronic renal failure.

A total of 16 trials [22, 24–36, 39, 40] reported the response rates of the treatment group as compared with the control group. There were 735 patients in the treatment group and 688 in the control group. The meta-analysis showed that there were significant higher response rates on Shen shuaining group comparing to the control group in the fixed effects model (Peto OR = 4.25, 95% CI: 3.32, 5.42, *P* < 0.00001), Figure 6.

### 3.7. Adverse Effects

Five studies [22, 29, 37, 39, 40] reported adverse reactions. The most common treatment emergent adverse effects were nausea/vomiting, and diarrhea, which were relieved after symptomatic treatment. Six studies [26, 31, 35, 36, 38, 41] mentioned that there was no adverse effect in both groups. The remaining trials did not report safety evaluation. So we did not conduct analysis on adverse effect because of the lack of further reports on severe side effects in all the clinical trials involved.

### 3.8. Sensitivity Analysis

The small number of studies within comparisons, and the lack of trials with low risk of bias prevented us from using sensitivity analysis or meta-analysis to have a further investigation of the heterogeneity.

### 3.9. Publication Bias

**Figure 7:**
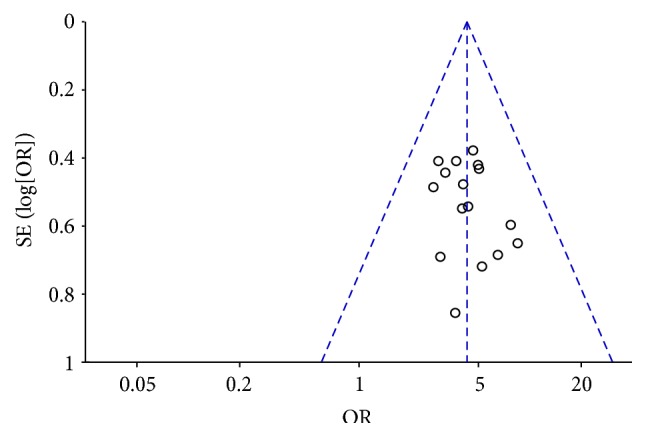
Funnel plot comparing treatment group to control group on response rate.

A funnel plot analysis of the 16 trials [22, 24–36, 39, 40] comparing treatment group to control group on response rate was generated to determine the potential publication bias, and it manifested an insignificant asymmetry in Figure 7.

## 4. Discussion

Although several clinical studies reporting Shen shuaining capsule for treating CKD patients ranged from case reports, case series, and controlled trials to randomized controlled trials, there was no systematic review specially dealing with its effectiveness in the treatment of CKD. So this is the first review to explore the efficacy of Shen shuaining capsule for CKD. A total of 20 RCTs involving 832 treatment patients and 774 control patients were identified for this review. Our results showed that the levels of BUN and SCR were significantly lower and the levels of HB and response rate were significantly improved in the treatment group compared with the control group, which suggested a protective effect of Shen shuaining capsule on renal function and an improvement on renal anemia in CKD patients. However, there is no evidence for increasing the CCR is more effective than the control group in CKD treatment. It might result from the small number of included trials on CCR. We divided the cases in the review into subgroups, according to intervention course in order to lower heterogeneity. Significant decreases on SCR, BUN levels were observed within Shen shuaining capsule after at least 4 weeks. Significant increases on Hb levels were observed within Shen shuaining capsule after at least 4 weeks. Nausea, vomiting, and diarrhea symptoms might occur after taking the capsule, but the symptoms were mild, and symptoms disappeared when medication was adjusted to the postprandial period. No studies indicated that patients discontinued treatment because of side effects. we could not conduct analysis on adverse effect because of the lack of further reports on severe side effects in all the clinical trials involved. However, there was no evidence to show that the experimental group was better than the control group in increasing the CCR.

The main findings revealed that Shen shuaining capsule in the treatment of CKD may increase renal function, response rate, and hemoglobin.

Several limitations should be realized when we accepted the findings of this meta-analysis. Although the publishing language was not limited, all studies were written in Chinese and conducted in China. Therefore, a location bias should be considered because of searching strategy restrictions. Most of the included studies were prone to some methodological issues and potential risk of bias. The quality of reporting in general was poor with few trials reporting the detailed random methods, allocation concealment, and level of blinding. There might be a high risk of selection bias with unclear randomization and allocation concealment, although all trials were reported baseline comparability. The possibility of performance bias and detection bias would increase as a result of lacking of blind method (at least for the patients and possibly also for others involved in the trial) and lacking of descriptions of dropouts/loss to follow-up and intentional analysis. And the poor reporting on side effects in several included trials limit the exploration of safety of Shen shuaining capsule. Second, heterogeneity may be another problem in this meta-analysis. Several factors like primary diseases of CKD, the degree of CKD, setting, and dosage might lead to heterogeneity. Publication bias might be serious in studies of TCM. A funnel plot analysis showed that there was potential publication bias in this meta-analysis. Moreover, we could not obtain unpublished studies from the manufacturer. In addition, the included 20 trials assessed the clinical curative effect using intermediate indicators (such as renal function, CCR, and Hb) lacking of endpoints: such as mortality rate, survival quality, and occurrence of end-stage renal failure into dialysis. So we could not determine the overall and long-term curative effect. And we also could not evaluate the effect of Shen shuaining capsule on the electrolyte balance, hypertension, and the quality of life for lack of relevant reports in the included trials.

Due to the possible limitations presented in this review, evidence for its effectiveness is needed to be testified in next step and recommendations for clinicians should be cautious. With regard to the small number of studies within comparisons, and lack of trials with low risk of bias, further well-designed randomized controlled trials are required to explore the effectiveness and safety of Shen shuaining capsule for patients with different degrees of CKD and various types of protopathy.

## 5. Conclusion

The current evidence suggests that the use of Shen shuaining capsule may better improve chronic renal failure control than Western medicine or routine conventional treatment. Shen shuaining capsule appeared to be effective for treating CKD. In particular, when duration of treatment reached more than 4 weeks or at least 4 weeks. The levels of SCR, BUN, and Hb were improved significantly. However, given the poor quality of the available evidence and high heterogeneity of the study results, further high quality, large trials using standardized preparation are warranted to better elucidate the effects of Shen shuaining capsule on CKD control.

## Figures and Tables

**Table 1:** Characteristics of included studies.

Study	*n* (T/C)	Baseline	Intervention measures		Course (week)	Outcome measures	Assessment of methodological quality						
			Experiment group	Control group			Random sequence generation	Allocation concealment	Blinding of participants and personnel outcome assessor	Blinding of outcome assessor	Incomplete outcome data	Selective reporting	Other sources of bias
Chen 2001 [22]	26/26	Accordance	Shen shuaining capsule + RT	Coated Aldehyde Oxystarch capsule + RT	3	⑤	Unclear	Unclear	Unclear	Unclear	Yes	Unclear	Unclear
Feng 2006 [23]	21/19	Accordance	Shen shuaining capsule + RT	Coated Aldehyde Oxystarch capsule	4	① ②	Unclear	Unclear	Unclear	Unclear	Yes	Unclear	Unclear
Lin 2007 [24]	45/41	Accordance	Shen shuaining capsule + RT	RT	6	① ② ④ ⑤	Unclear	Unclear	Unclear	Unclear	Yes	Unclear	Unclear
Zhou 2012 [25]	56/56	Accordance	Shen shuaining capsule + RT	RT	6	⑤	Unclear	Unclear	Unclear	Unclear	Yes	Unclear	Unclear
Wang 2013 [26]	24/24	Accordance	Shen shuaining capsule + RT	Coated Aldehyde Oxystarch capsule	6	⑤	Unclear	Unclear	Unclear	Unclear	Yes	Unclear	Unclear
Xu 2013 [27]	30/30	Accordance	Shen shuaining capsule + RT	Coated Aldehyde Oxystarch capsule	8	① ② ⑤	Unclear	Unclear	Unclear	Unclear	Yes	Unclear	Unclear
Wu 2014 [28]	66/50	Accordance	Shen shuaining capsule + RT	RT	6	① ② ③ ④ ⑤	Stratified random	Unclear	Unclear	Unclear	Yes	Unclear	Unclear
Wu 1998 [29]	60/56	Accordance	Shen shuaining capsule + RT	Coated Aldehyde Oxystarch capsule	6	① ② ④ ⑤	Unclear	Unclear	Unclear	Unclear	Yes	Unclear	Unclear
Huang and Wang 2000 [30]	60/60	Accordance	Shen shuaining capsule + RT	RT	6	① ② ⑤	Unclear	Unclear	Unclear	Unclear	Yes	Unclear	Unclear
Yang 2003 [31]	30/32	Accordance	Shen shuaining capsule + RT	RT	6	① ② ⑤	Unclear	Unclear	Unclear	Unclear	Yes	Unclear	Unclear
Xu 2007 [32]	56/56	Accordance	Shen shuaining capsule + RT	Medicinal Charcoal Capsule	8	⑤	Unclear	Unclear	Unclear	Unclear	Yes	Unclear	Unclear
Wang 2009 [33]	68/68	Accordance	Shen shuaining capsule + RT	Coated Aldehyde Oxystarch capsule	6	⑤	Unclear	Unclear	Unclear	Unclear	Yes	Unclear	Unclear
Zhan 2002 [34]	45/31	Accordance	Shen shuaining capsule + RT	Coated Aldehyde Oxystarch capsule	24	① ② ③ ⑤	Unclear	Unclear	Unclear	Unclear	Yes	Unclear	Unclear
Liu 2009 [35]	50/50	Accordance	Shen shuaining capsule + RT	RT	12	① ② ⑤	Unclear	Unclear	Unclear	Unclear	Yes	Unclear	Unclear
Zhang 2011 [36]	46/32	Accordance	Shen shuaining capsule + RT	Coated Aldehyde Oxystarch capsule	24	⑤	Unclear	Unclear	Unclear	Unclear	Yes	Unclear	Unclear
Wang 2003 [37]	30/30	Accordance	Shen shuaining capsule + RT	RT	48	① ③ ④	Unclear	Unclear	Unclear	Unclear	Yes	Unclear	Unclear
Kang 2009 [38]	22/21	Accordance	Shen shuaining capsule + RT	RT	12	① ②	Random number of tables	Unclear	Unclear	Unclear	Yes	Unclear	Unclear
Yao 2014 [39]	42/42	Accordance	Shen shuaining capsule + RT	RT	24	⑤	Unclear	Unclear	Unclear	Unclear	Yes	Unclear	Unclear
Zhang 2014 [40]	30/30	Accordance	Shen shuaining capsule + RT	RT	48	⑤	Unclear	Unclear	Unclear	Unclear	Yes	Unclear	Unclear
Liu 2007 [41]	25/20	Accordance	Shen shuaining capsule + RT	Coated Aldehyde Oxystarch capsule	6	① ② ④	Unclear	Unclear	Unclear	Unclear	Yes	Unclear	Unclear

RT: routine conventional treatment.

① SCR; ② BUN; ③ CCR; ④ Hb; ⑤ response rate.

Yes: low risk of bias; no: high risk of bias; unclear: uncertain risk of bias.
